# Development of Antimicrobial Therapy Methods to Overcome the Antibiotic Resistance of Acinetobacter baumannii

**DOI:** 10.32607/actanaturae.10955

**Published:** 2020

**Authors:** O. V. Kisil, T. A. Efimenko, N. I. Gabrielyan, O. V. Efremenkova

**Affiliations:** Gause Institute of New Antibiotics, Moscow, 119021 Russia; V.I. Shumakov Federal Research Center of Transplantology and Artificial Organs, Ministry of Healthcare of the Russian Federation, Moscow, 1123182 Russia

**Keywords:** Acinetobacter baumannii, multidrug resistance, biofilms, bacteriophage therapy, antimicrobial peptides

## Abstract

The spread of antibiotic resistance among pathogens represents a threat to human health around the world. In 2017, the World Health Organization published a list of 12 top-priority antibiotic-resistant pathogenic bacteria for which new effective antibiotics or new ways of treating the infections caused by them are needed. This review focuses on *Acinetobacter baumannii*, one of these top-priority pathogens. The pathogenic bacterium *A. baumannii *is one of the most frequently encountered infectious agents in the world; its clinically significant features include resistance to UV light, drying, disinfectants, and antibiotics. This review looks at the various attempts that have been made to tackle the problem of drug resistance relating to *A. baumannii *variants without the use of antibiotics. The potential of bacteriophages and antimicrobial peptides in the treatment of infections caused by *A. baumannii *in both planktonic and biofilm form is assessed. Such topics as research into the development of vaccines based on the outer membrane proteins of *A. baumannii *and the use of silver nanoparticles, as well as photodynamic and chelate therapy, are also covered.

## INTRODUCTION

Antimicrobial therapy is among the most consequential medical breakthroughs achieved in the 20th century. It has helped save millions of lives. However, antimicrobial therapy also has shortcomings, such as a certain degree of toxicity, microbiome disturbance, and the formation of resistant pathogen forms causing serious infectious diseases. Their rapid spread threatens to dent the effectiveness of modern medicine, including that of surgical intervention, organ transplantation, and hematologic diseases when patients have a weakened immune system and, therefore, the risk of infection increases. According to the World Health Organization (WHO), *Acinetobacter baumannii *is one amongst six particularly dangerous bacteria because it is multidrug-resistant (MDR) and does not respond to antimicrobial therapy. For these bacteria, WHO has suggested using the abbreviation ESKAPE (to *escape *from the action of antibiotics): *Enterococcus faecium, Staphylococcus aureus, Klebsiella pneumoniae, Acinetobacter baumannii, Pseudomonas aeruginosa, *and *Enterobacter *spp*. *[1]. After eight years, the list of bacterial pathogens that do not respond to antimicrobial therapy was expanded to 12 and the bacteria were subdivided into three groups according to their level of threat to human health (critical, high or medium); new effective antibiotics or new ways to treat infections caused by these pathogens need to be developed [2].

Numerous articles published thus far have suggested various options for antimicrobial therapy that are effective on the infections caused by these pathogens [3]. Our review focuses exclusively on antibiotic-resistant strains of the Gram-negative *A. baumannii *pathogen and aims to describe alternative approaches to the treatment of infections caused by *A. baumannii*, including bacteriophage therapy, preventive vaccination, light therapy, silver ion therapy, and chelate therapy.

**Fig. 1:**
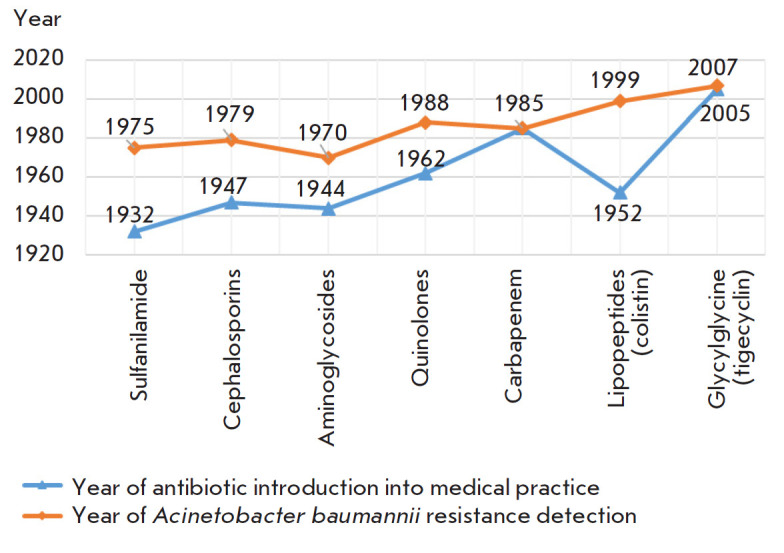
Time intervals between the introduction of an antibiotic into medical practice and the first reports of *Acinetobacter baumannii *resistance [5]

The genus *Acinetobacter *contains Gram-negative, strictly aerobic, lactose-fermenting, fixed rod-shaped bacteria. Members of the genus *Acinetobacter *are ubiquitous saprophytic microorganisms. They can be isolated from various sources: soil, surface water, and the mucous membranes of the upper respiratory tract of humans. The genus *Acinetobacter *currently includes 27 species. From a clinical point of view, three phylogenetically related *Acinetobacter *species are of the greatest interest: *A. baumannii, A. pittii*, and *A. nosocomialis*. They are the most significant pathogens causing nosocomial infections [4]. The important adaptive features of *A. baumannii *include its high mutation rate, which leads to rapid development of antibiotic resistance. *Figure 1* shows the time intervals separating the introduction of an antibiotic into medical practice and the detection of resistance by *A. baumannii *to it [5].

Presumably, the first infections caused by *A. baumannii *were documented at U.S. military treatment facilities during the wars in Iraq and Afghanistan [6, 7]. *Acinetobacter baumannii *was even referred to as “Iraqibacter”, since it affected thousands of American soldiers during the Iraq war [8]. The first studies of hospital-acquired infections caused by *A. baumannii *were conducted in the early 1980s [9, 10]. It is interesting to note that 30 years ago infections caused by *Acinetobacte*r species were not considered a public health threat, although the mechanisms of innate resistance by *A. baumannii *were documented and described. However, the research conducted over the past decade has shown that in addition to its own internal resistance mechanisms, *A. baumannii *can successfully acquire multiple determinants of resistance by horizontal gene transfer, becoming an MDR bacterium. Today, *A. baumannii *MDR strains are endemic and epidemic in hospitals around the world, with mortality rates ranging from 40% to 70% for diseases requiring artificial lung ventilation, 25–30% for meningitis, and 34–49% for bacteremia [11]. A study of infections spread in intensive care units conducted in 75 countries across five continents assumes that *A. baumannii *is one of the most common infectious agents in the world [12]. The WHO estimates that the spread of MDR *A*. *baumannii *is today a serious global threat. *Table 1*shows the main stages in recognizing *A. baumannii* as a multidrug-resistant nosocomial pathogen.

Sequencing of the genomes of 49 strains of MDR *A. baumannii *within one U.S. hospital system showed that almost every analyzed strain was unique [25]. A comparative analysis of *A. baumannii *strains revealed a transfer of mobile genetic elements, homologous recombination within the entire genome, deletions and mutations, all occurring within short periods of time. The variations in the gene composition of the strains did not have clear spatial (location in a hospital) or temporal patterns, thus proving that there was a pool of circulating strains in this hospital with significant interstrain interaction. Thus, the exchange of genetic material and rearrangements of the bacterial genome lead to multiple genetic combinations and provide an infinite source of genetic adaptability for *A. baumannii. *

**Fig. 2:**
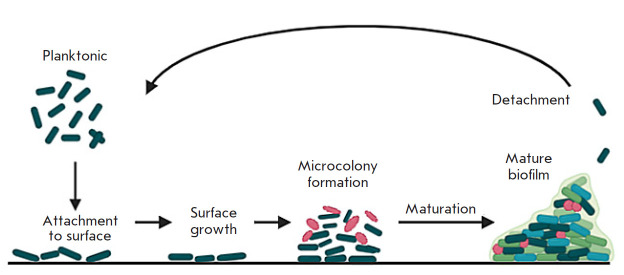
Stages of biofilm formation

*A. baumannii *is a successfully survivable in-hospital pathogen not only because of its ability to “switch” its genomic structure and capture resistance markers, but also because of its innate biofilm-forming ability [11]. In contrast to the planktonic state, biofilms are communities of bacteria enclosed in a self-produced exopolysaccharide matrix that serves to attach the bacteria to surfaces, including medical implants and human tissue: teeth, skin, trachea, and urethra. It is known that bacteria in the biofilm can be 10–1,000 times more resistant to antibiotics than their planktonic forms [26]. Infections associated with the formation of biofilms attached to surfaces are very difficult to treat. Therefore, preventing the early stage of biofilm formation is considered an important step in infection prevention and treatment.

Biofilm formation is a step-by-step process that includes three phases: adhesion, maturation, and detachment (*Fig. 2*). During the adhesion phase, plankton cells attach to the surface through weak interactions [27]. After initial attachment, weakly bound cells stably attach due to more specific molecular interactions between bacterial surface structures such as pili and host molecules functioning as receptors (such as fibronectin). During the biofilm maturation phase, bacteria produce large amounts of exopolysaccharides, which form most of the biofilm’s biomass. During the detachment phase, cells (single or clusters) separate and colonize neighboring sites. The biofilm is highly resistant to drugs because of the low diffusion of antibiotics in it, the presence of persistent cells, and the slow growth rates and low metabolism of cells deep in the biofilm. Due to the proximity of the cells, the biofilm is characterized by increased horizontal transfer of resistance genes. It has been proved that *A. baumannii *can attach to tissues and form a biofilm at a surgical site, which complicates infection prevention and treatment and is especially critical when medical implants are used [28].

**Table 1:** Historical reference of the Acinetobacter baumannii pathogen

Year	Fact	Reference
1911	The genus Acinetobacter was first described	[13]
1968	The modern designation of the genus Acinetobacter (from the Greek akinetos, “fixed”) proposed by Brisou and Prevot in 1954, was accepted.	[14, 15]
1974	The genus Acinetobacter designation is included in Bergey’s Manual of Systematic Bacteriology (described as having only one species: Acinetobacter calcoaceticus)	[16]
1984	First report of resistance to imipenem	[17]
1986	The Acinetobacter calcoaceticus-baumannii complex is divided into four species based on DNA hybridization studies: A. calcoaceticus; A. baumannii; A. pittii; A. nosocomialis A. baumannii is described as an agent that causes a nosocomial infection	[18]
1999	First report of resistance to colistin	[19]
2001	The WHO published the first international appeal: “Global Strategy for Containment of Antimicrobial Resistance”	[20]
2007	First report of resistance to tigecycline	[21]
2009	Bacteria that are dangerous to human health are grouped in ESKAPE (including Acinetobacter)	[1]
	The USA (CDC) and EU (ECDC) established the Transatlantic Taskforce on Antimicrobial Resistance (TATFAR)	[22]
2015	The WHO developed a new “Global Strategy for Containment of Antimicrobial Resistance”	[23]
2017	The WHO published the “Global priority list of antibiotic-resistant bacteria to guide research, discovery, and development of new antibiotics”	[24]

During outbreaks of nosocomial infections, *A. baumannii *isolates have been found on various surfaces surrounding patients, including furniture and hospital equipment, doors, electrical switches, wash basins, etc. (over 30 items) [11]. It is noteworthy that outbreaks associated with infected items have ended once the source of the infection was removed, replaced, or properly disinfected. Today, proper hygiene, and hand hygiene in particular, is an efficient and simple means for preventing a bacterial infection of whatever nature.

The mechanism of *A. baumannii *infection is associated with a number of factors, including a long hospital stay (especially in intensive care units), the disease severity, blood transfusion, the use of an intravascular catheter or endotracheal tube, intubation with artificial ventilation, inadequate initial antibacterial therapy, and contamination of patient environment with *A. baumannii. *Contaminated surfaces, medical equipment, poor hand hygiene, and violations of sanitary requirements by patients and medical staff can be the cause of infection and result in its rapid transmission; medical staff transmits microorganisms to patients or facilitates bacteria exchange between patients [29]. *A. baumannii *is transmitted from person to person through airborne droplets: so, the respiratory system is the main infection route. Kotay et al. [30] found that bacteria can also spread through wash basins. It was shown that the bacteria, in the form of a biofilm, multiply in drain pipes and gradually occupy the space higher up the pipe towards the wash basin. Water flows from a faucet lead to dispersion of droplets, which spread the bacteria.

Diseases caused by *A. baumannii *do not differ in any special clinical manifestations from other infections. However, the following specific features may help medical staff determine whether a patient is infected with *A. baumannii*: (1) late infection and (2) excessive use of broad-spectrum antibiotics in the early stages of treatment. The loose use of antibiotics is considered the main reason behind the development of a significant proportion of MDR *A. baumannii *variations [31]. It has been repeatedly shown that administration of antibiotics in concentrations below MIC increases the probability of *A. baumannii *biofilm formation [32].

The effectiveness of antimicrobial drugs against Gram-negative bacteria depends on the balance between several fundamental molecular intracellular processes that occur before the antimicrobial drug interacts with the target: (1) drug influx mediated by porins; (2) drug outflow mediated by efflux systems; (3) drug inactivation, usually by irreversible cleavage catalyzed by periplasmic and cytoplasmic enzymes; and (4) modification of the target to which the drug can bind [33]. High antimicrobial resistance of *A. baumannii *is due to an interconnection between all these mechanisms. It is achieved by obtaining new genetic information through horizontal gene transfer and mutations. New genetic determinants are acquired by *A. baumannii *strains through the combined effect of mobile genetic elements (insertion sequences, transposons), integrons, and transferable plasmids. Changes can be a result of either spontaneous mutations leading to a modification of the drug target or insertions/deletions of the mobile elements that alter the expression of endogenous resistance mechanisms or membrane permeability. In addition to these mechanisms, *A. baumannii *can accumulate many determinants of resistance in the so-called “resistance islands” (specific genome regions containing clusters of horizontally transferred DNA that include antimicrobial resistance genes). Such clusters provide a “shelter” to mobile elements, since insertion into this site causes no damage to the host cell [34, 35]. It has been assumed that *Acinetobacter *spp*. *can play an important role in the transfer of resistance genes to other Gram-negative microorganisms [36].

## MATERIALS AND METHODS

Thirty years ago, infections caused by *A. baumannii *could be effectively treated with conventional antibiotics, but the global spread of MDR strains has dramatically reduced the number of agents that are effective on infections caused by this pathogen. To date, it has been established that *A. baumannii *is resistant to such antibiotics as penicillins, cephalosporins, chloramphenicol, aminoglycosides, fluoroquinolones, and tetracyclines [29]. Multidrug resistance of many clinical *A. baumannii *isolates severely restricts the currently available treatment options, so there is an urgent need for new therapies and methods that would be effective against MDR *A. baumannii*.

In recent years, combination therapy has been increasingly used for infections caused by MDR Gram-negative bacteria. It is obvious that the probability of resistance against a combination of two drugs is much less than that against one drug. In addition, the synergistic effect of combination antibiotics exceeds the effect of antibiotic monotherapy. However, some combinations cause an opposite effect, resulting in much more severe damage. One antibiotic can induce resistance to the second antibiotic administered within the combination, thus leading to an antagonistic effect [3].

Adjuvants show good prospects for use in clinical antibacterial practice. These substances per se have almost no antimicrobial activity, but in combination with antibiotics, adjuvants can inhibit resistance mechanisms in various ways: (1) by increasing antibiotic absorption through the bacterial membrane; (2) by inhibiting efflux pumps; and (3) by changing the physiology of resistant cells that promote biofilm spreading (in particular, by quorum quenching) [37]. It is known that bacteria produce the chemical signals necessary for intercellular communication and adaptation to the environment. The mechanism of quorum sensing in bacteria consists in the expression of a certain phenotype when a high population density is reached [38]. The molecules inhibiting quorum sensing suppress phenotypic manifestation of the trait, such as biofilm formation. Combinations of 1-[(2,4-dichlorophenethyl) amino]-3-phenoxypropan-2-ol and combinations with various antibiotics inhibit the growth of all pathogens of the ESKAPE group in both planktonic and biofilm form [39].

The number of antibiotics effective on Gram-negative infections decreases with every year. In the 21^st^ century, only 33 antibiotics have been introduced into medical practice, including only two new natural antibiotics, daptomycin and fidaxomicin [40]. An analysis of the list of antibiotics recommended by the Clinical and Laboratory Standards Institute (CLSI, USA) has shown that since 2010, many antibiotics proposed for the treatment of ESKAPE-related infections have been replaced by a relatively small number of antibiotic + antibiotic combinations [3]. Thus, due to the limited availability of antibiotics for treating infections caused by Gram-negative MDR bacteria, alternative strategies are needed. Among them, feature such methods as the use of bacteriophages and their enzymes, antimicrobial peptides, photodynamic and chelate therapy, and nanoparticles.

**Bacteriophage therapy**

One of the possible therapeutic agents against *A. baumannii *is bacteriophages, the most widely encountered organisms on the planet, whose number exceeds 1031 according to a number of estimates [41]. The fundamental aspect of phage–bacterium interaction is phage specificity, i.e. the ability to infect a strictly defined host bacterium. Bacteriophages are adsorbed on the bacterial cell, inject their genome through the membrane into the cell, through which mechanism they express their own genes, replicate the genome in the host cell, and release virions after lysis of bacterial cells. The advantages of bacteriophage over antibiotic therapy include drug tolerance and the fact that bacteria develop resistance to bacteriophages at the lowest rate. In addition, bacteriophages are highly specific to their targets, unlike broad-spectrum antibiotics, which kill normal bacterial flora and disrupt the microbiome of healthy humans [42].

As the incidence and mortality rate of MDR pathogens increase, interest in bacteriophages is returning all over the world. Since 2010, scientists from different countries have discovered new bacteriophages infecting MDR *A. baumannii *[43-46]. In most cases, bacteriophages against *A. baumannii *have been studied *in vitro*, but the ability of bacteriophages to lyse *A. baumannii *has recently increasingly come to be evaluated by simulating the infectious process *in vivo*. *Table 2* summarizes the results of bacteriophage therapy of infections caused by *A. baumannii *over the past five years. Thus, it was shown that two lytic bacteriophages isolated from hospital wastewater were able to infect more than 50% of carbapenem-resistant clinical strains of *A. baumannii*. Less than 20% of *Galleria mellonella *larvae survived 96 h after infection with *A. baumannii. *With the introduction of bacteriophages, larval survival increased to 75%, while treatment with polymyxin B increased survival to only 25% [47]. Improvement in wound infection healing in the phage-infected group and a significant reduction in mortality in rats, compared to infected animals treated with an antibiotic, was also observed [48].

**Table 2:** Summary of the data from studies on bacteriophage use

Antimicrobial agent	Infection model	Efficiency of infection inhibition	Antibiofilm activity	Reference
WCHABP1, WCHABP12	Larvae of Galleria mellonella infected by A. baumannii	The survival of larvae of Galleria mellonella increased to 75%	*	[47]
Phage (without definition, probably belongs to the Siphoviridae family)	Rat wound infection	100% inhibition of the pathogen	*	[48]
Cocktail of AB-Army1 and AB-Navy1-4	Murine wound infection	Inhibition of the pathogen	▲	[49]
Cocktail of AB-Navy1, AB-Navy4, AB-Navy71, AB-Navy97 and AbTP3Φ1	Human pancreatic pseudocyst	100% inhibition of the pathogen	*	[50]

Note: “*” – no data; “▲” – biofilm destruction.

A bacteriophage cocktail was successfully used against *A. baumannii *in the mouse model of a full-thickness dorsal infected wound: bacterial load in the wound decreased, thus preventing the spread of the infection and necrosis in surrounding tissues [49]. It was shown that the bacteriophages in the cocktail function in combination: the action of one of them is aimed at transferring the population of *A. baumannii *from the biofilm to the planktonic state, in which the cells are sensitive to other bacteriophages in the mixture. Although individual bacteriophages in that study exhibited some antibacterial properties, they were not as effective as a complex bacteriophage cocktail [49]. It should be noted that testing a phage cocktail against a collection of 92 clinical isolates of MDR *A. baumannii *revealed that only 10 strains were susceptible to therapy: this fact emphasizes that the spectrum of action of phages is very narrow, which must be taken into account when using them as therapeutic agents. So, it is optimal to use bacteriophages belonging to different families and having a wide range of hosts (different *A. baumannii *isolates) to prepare a phage cocktail.

A bacteriophage cocktail was successfully applied in the treatment of a diabetic patient with necrotizing pancreatitis complicated by a MDR *A. baumannii *infection [50]. Despite numerous courses of antibiotics (a combination of meropenem, tigecycline, and colistin), the condition of the 68-year-old patient deteriorated over a 4-month period. After the failure of antibiotic treatment, three phage cocktails with lytic activity against *A. baumannii *were prepared. Administration of these bacteriophages intravenously and percutaneously into the abscess cavities led to complete cure of the patient. It should be noted that during bacteriophage therapy, serial *A. baumannii *isolates with significantly reduced sensitivity to the introduced phages appeared; i.e., the *A. baumannii *population started to evolve in response to the selection pressure exerted by the phages. This aligns with the data [51] showing that during the use of bacteriophages some *A. baumannii *can acquire resistance and avoid lysis by bacteriophages. A bacteriophage loses its ability to effectively infect its bacterial host if receptors become unavailable, for example, due to the biofilm formation that prevents bacteriophage access to the cell membrane. Although the bacteriophage cocktail had lost its antibacterial activity, it still prevented the growth of *A. baumannii *with increased resistance to minocycline [50]. This antibiotic was added to bacteriophage therapy 4 days after the initial administration of the cocktail. The combinatorial activity existing between bacteriophages and conventional antibiotics was previously demonstrated in animal models [49]. Once the *A. baumannii *population is transferred to an encapsulated state, antibiotics can more readily penetrate the bacterial membrane. Thus, in addition to potential therapeutic applications, bacteriophages can be used to eliminate *A. baumannii *biofilms. In this case, the combination of phages with antibiotics creates a situation in which bacteria are destroyed either by the bacteriophage, or by an antibiotic, or through their combined action.

It is assumed that bacteriophages can transfer the genetic elements that cause drug resistance and pathogenicity in bacteria. However, culturing on a bacterial isolate already present in the patient minimizes the risk of introducing exogenous genetic information that ensures increased virulence or resistance to antibiotics. In addition, the natural specificity of a bacteriophage to a bacterial type and even strain minimizes the potential for horizontal gene transfer, compared to more random plasmid conjugation or absorption of exogenous DNA in nature.

The numerous advances achieved in the treatment of MDR *A. baumannii *infections through local and systemic administration of bacteriophages, including in combination with antibiotics, highlight the potential of bacteriophages as relates to bacterial infections. However, bacteriophage therapy is difficult to standardize for mass production. In addition, the complete genomes of bacteriophages contain some genes with unknown functions: so, it is difficult to predict the long-term safety of bacteriophages [52].

Phage adsorption on a susceptible host cell is determined by a specific interaction between the phage’s receptor-binding proteins located on the tail fibrils (with or without enzymatic activity) and a specific receptor on the cell surface. Exopolysaccharide depolymerases are responsible for partial destruction of the exopolysaccharides of the bacterial cell wall. These enzymes are shared components between bacteriophage spines and fibrils. Destruction of the bacterial capsule reduces biofilm formation and, as a result, antibiotic resistance: so, using bacteriophage depolymerases to eliminate the biofilm in the treatment of bacterial infections was proposed [53, 54, 55]. Various isolated phages against *A. baumannii *were shown to encode depolymerase, which successfully eliminates the capsular exopolysaccharide of the bacterium [53, 56, 57]. Thus, endolysin (LysAB3) of phage φAB3 specific to *A. baumannii *effectively eliminates the biofilm associated with *A. baumannii in vitro *[58]. The antibacterial mechanism of LysAB3 may be associated with the ability of the structural region of amphiphilic peptide to enhance the permeability of the cytoplasmic membrane of *A. baumannii *by degradation of bacterial wall peptidoglycan.

Bacteriophages infecting *Acinetobacter *species are usually highly specific to the host strain [59]. From the perspective of therapeutic application, the high specificity of bacteriophages can be considered as either a useful or a limiting factor. However, if the genes encoding the bacteriophage’s fibril tail protein are replaced with genes from other phages, the new chimeric phage will lose its sensitivity to the original hosts and be able to lyse the new hosts. Thus, the chimeric phage φAB1tf6 obtained by replacing the gene encoding the tail fiber protein of phage φAB1 with the corresponding gene from φAB6 has acquired the host range of the second bacteriophage [53].

## EXPERIMENTAL

The bacteriophage’s tail spine proteins can be used as a bioengineering tool to obtain a glycoconjugate vaccine against *A. baumannii *[53, 60, 61]. Glycoconjugate vaccines are produced by conjugating an antibacterial exopolysaccharide to a carrier protein. The vaccine, based on oligosaccharide fragments, elicits a stronger immune response compared to that elicited by a vaccine based on whole bacterial exopolysaccharides, due to their heterogeneity. Chemical synthesis of polysaccharides is labor-intensive and has a low yield, while chemical hydrolysis of bacterial exopolysaccharides yields a mixture of heterogeneous oligosaccharide fragments. Using bacteriophage tail spine proteins that can hydrolyze the bacterial exopolysaccharide is a potential alternative to obtaining oligosaccharides of a given size. It has been shown that the tail spike protein of bacteriophage φAB6 can depolymerize the exopolysaccharide of the *A. baumannii *strain 54149, with the formation of homogeneous oligosaccharide fragments that can be used as a platform for obtaining a glycoconjugate vaccine [60, 61].

Prophylactic vaccination can be one of the alternative methods to combat bacterial infections [62]. A classic vaccine is a pharmaceutical product that stimulates the immune system, thus preventing pathogen development. To trigger a long-term immune response that includes both the innate and adaptive immune systems, the vaccine must resemble the pathogen but not cause the concomitant disease. In the initial developments of vaccines against *A. baumannii, *it was assumed that a lot of bacterial antigens must be included in the vaccine. It was believed that whole-cell vaccines could stimulate a response against multiple antigens, which would provide protection against a wide range of strains within a species. Thus, outer membrane vesicles of *A. baumannii *were successfully used as an antigen [63]. The inactivated whole-cell vaccine successfully protected mice against two clinical isolates of *A. baumannii*, including a resistant strain. Later, separate bacterial components were used to develop the vaccine. It was discovered using a murine model that vaccination with a specific cell surface protein involved in the formation of a *A. baumannii *biofilm reduces the bacterial load in tissues and ensures high antibody titers [64].

The *A. baumannii *outer membrane proteins OmpA, Omp34 kDa, and OprC were shown to be effective in developing an antibacterial vaccine. A DNA vaccine consisting of plasmids encoding two proteins of the *A. baumannii *outer membrane, OmpA and Pal, was designed [65]. The OmpA protein is considered the most promising antigen for developing vaccines against *A. baumannii*, since it is a virulence factor involved in the pathogenesis of *A. baumannii *and shows high immunogenicity in animal models. In addition, OmpA is highly conserved among various strains; it is the most common protein identified in the outer membrane vesicles of *A. baumannii*. Pal is a peptidoglycan-associated cell wall lipoprotein that plays an important role in ensuring outer membrane integrity. A mouse model of pneumonia showed the significant efficacy of the DNA vaccine against an acute *A. baumannii *infection; effective cross-protection was observed when we immunized mice infected with clinical strains of *A. baumannii*.

Prophylactic vaccination and passive immunization can be very effective tools in preventing and treating the most common and serious infections caused by *A. baumannii*. However, only a few vaccines tested on animals have been included in clinical studies, and no vaccine against *A. baumannii *has yet been approved for human vaccination. In addition, the question remains: which population groups will benefit from prophylactic vaccination against *A. baumannii *and when should they be vaccinated?

**Antimicrobial peptides**

Antimicrobial peptides (AMPs) meet the definition of “antibiotics.” They are formed by living organisms and exhibit an antibiotic effect against pathogens. One of the first antibiotics was lysozyme isolated from human tears and saliva by Alexander Fleming in the 1920s. In 1939, at the beginning of antibiotics science, gramicidins, peptide antibiotics of bacillary origin, were described. AMPs are now found in organisms belonging to all taxonomic groups. In most multicellular organisms, AMPs are the central element of the non-specific innate defense system; it is the first line of defense against an invasion by a wide range of pathogens [66-68]. This review considers a special group of antimicrobial peptides; namely, those formed in the human and animal bodies. These AMPs also meet the definition of “humoral factors of innate immunity” [69].

Natural antimicrobial peptides usually consist of 12–60 amino acid residues and contain cationic amino acids, usually arginine and lysine residues. This allows AMPs to interact with negatively charged bacterial membranes and, in some cases, even penetrate them (translocate into host cells) due to a large electric potential gradient, which leads to bacterial cell lysis [70]. In addition to destroying the membranes, AMPs can interfere within intracellular processes, preventing the biosynthesis of nucleic acids, proteins, and cell walls. Furthermore, cell wall peptidoglycans, cytosolic RNAs, proteins, and cytosolic enzymes/chaperones can act as targets for AMPs [71].

**Table 3:** Summary of the data from studies of the use of antimicrobial peptides (AMPs)

Antimicrobial agent	Infection model	Efficiency inhibition of the infection	Antibiofilm activity	Reference
Histatin 5 (N)	In vitro	85–90% inhibition of the pathogen	–	[73]
LL37 (N), WLBU2 (S)	In vitro	Inhibition of the pathogen	Δ	[28]
1018 (N)	In vitro	Inhibition of the pathogen	▲, Δ	[74]
HBcARD-150-177C (M)	Mouse model of lung infection	The survival of mice increased to 62.5–80%	*	[75]
SAAP-148 (S)	Ex vivo mouse and in vivo human wound skin infection	100% inhibition of the pathogen	▲, Δ	[76]
К11 (S)	Murine wound infection	99% inhibition of the pathogen	*	[77]
N10 (S), NB2 (S)	In vitro	Inhibition of the pathogen	▲	[79]

Note: “N” – naturally occurring AMPs; “M” – modification of naturally occurring AMPs; “S” – synthetic AMPs; “–” – no activity; “*” – no data; “▲” – biofilm destruction, “Δ” – prevention of biofilm formation.

Today, many of the AMPs of higher organisms are undergoing clinical trials as potential new antimicrobials, or as adjuncts to existing antibiotics in treatment regimens for infectious diseases [72]. *Table 3* summarizes the results of a study of the ability of AMPs to inhibit infections caused by *A. baumannii*. Histatin 5 (Hst 5), a histidine-rich AMP isolated from human and higher primate saliva, was shown to exhibit strong bactericidal activity against ESKAPE pathogens [73]. The action of this AMP caused the death of 85–90% of *A. baumannii *cells, while Hst 5 showed no significant antibiofilm activity. Conjugation of Hst 5 with spermidine was found to increase the bactericidal activity of the peptide against *A. baumannii*. The results of testing of the natural peptide 1018 triggering the degradation of the important signaling nucleotide (p)ppGpp have been reported [74]. Treatment with peptide 1018 at concentrations having no effect on plankton cell growth fully prevented the formation of biofilms and led to the destruction of mature biofilms in representative strains of both Gram-positive and Gram-negative pathogens, including *A. baumannii. *Low concentrations of peptide 1018 led to biofilm dispersal; higher concentrations caused the death of biofilm cells. Thus, the recognition and dispersal of bacterial membranes (without destroying the bacteria) can interfere with bacterial attachment to surfaces (such as medical implants or surgical sites) and contribute to the success of antimicrobial therapy.

In addition to natural AMPs, synthetic derivatives with improved activity have been proposed; natural AMPs were used as a reference template for their development. Chimeric AMPs created from two different AMPs were shown to improve antimicrobial activity. Other successful examples of AMPs modification include substitutions with D-amino acids, β-naphthylalanine, and α,α-dialkyl amino acids [75]. A panel of synthetic peptides was obtained based on human LL-37 AMP [76]. It was shown that peptide SAAP-148 suppresses MDR *A. baumannii *without causing resistance and prevents biofilm formation. A 4-h course of treatment with a hypromellose ointment containing SAAP-148 was shown to completely eliminate acute and biofilm-related *A. baumannii *infections in an *ex vivo *human wound infection model and an *in vivo *murine skin infection model. Synthetic peptide K11 (a hybrid of melittin, cecropin A1, and magainin 2) in subinhibitory concentrations exhibits antimicrobial activity against *A. baumannii *[77]. In addition, K11 can modulate oxidant and antioxidant levels, thereby promoting wound tissue regeneration in mice. K11 mixed with carbopol hydrogel heals infected wounds thanks to the synergism of the antibacterial properties of AMP and the moisturizing properties of the gel. Thus, thanks to their dual bioactivity, AMPs can destroy an infection and simultaneously exhibit immunomodulatory properties. Therefore, AMPs are considered a promising therapeutic tool for treating skin and soft tissue infections.

The phage display technique is one of the approaches used to identify peptides with antibacterial properties [78]. This method was used to select peptides targeted to *A. baumannii *[79]. To search for antimicrobial peptides against *A. baumannii *growing either in planktonic or biofilm form, biopanning was performed using a peptide library on five XDR *A. baumannii *strains grown in a medium containing human blood (blood biopanning) and the biofilms formed by these strains (biofilm biopanning). Thus, a number of peptides specific to *A. baumannii *were detected. Among those, two peptides were selected based on the similarity of their amino acid composition to that of other known AMPs. Both peptides exhibited antibacterial activity against *A. baumannii *(MIC 500 μg/mL), as well as significant antimicrobial activity; the combination of these two peptides more effectively reduced the formation of a *A. baumannii *biofilm compared to each individual peptide [79].

However, despite the numerous successful results both *in vitro *and *in vivo*, new AMPs have not found clinical application, yet. Destruction of AMPs by tissue proteases and their cytotoxicity stands in the way of their introduction into clinical practice.

**Antimicrobial photodynamic therapy**

Antimicrobial photodynamic therapy, either per se or in combination with a photosensitizer, induces photooxidative stress, which causes microbial death. *In vitro *studies have shown that blue light is effective against both planktonic and biofilm-growth forms of all six ESKAPE pathogens, including *A. baumannii *[80]. This conclusion has also been confirmed through *in vivo *data. It was shown that the use of weakly penetrating blue light (λ = 415 ± 10 nm) may be preferable for wound infections and the disinfection of a hospital environment. Bacterial biofilms were also highly susceptible to blue light. In general, antimicrobial photodynamic therapy is a promising approach to treating infections caused by ESKAPE pathogens, especially when applied topically.

## EXPERIMENTAL PROCEDURES

**Metal nanoparticles**

Metal nanoparticles, especially silver and silver-containing compounds, have recently been of increasing interest for managing bacterial infections. Silver nanoparticles (AgNPs) synthesized using physical, chemical, or biological methods release silver cations that disrupt electron transport and signal pathways or cause the formation of reactive oxygen species, which ultimately damage important biomolecules such as cell wall components, membranes, DNA, or proteins. Silver is an effective low-toxicity antimicrobial agent. A combination of AgNPs and antibiotics may be an effective solution to the problem of MDR *A. baumannii*; they can possibly be used at lower and less toxic doses compared to the drugs currently commonly used in clinical settings. In mice infected with carbapenem-resistant *A. baumannii*, synergistic antibacterial activity of AgNPs, in combination with polymyxin B, was detected; the survival rate was 60% compared to the control group receiving the antibiotic or AgNP alone [81]. Cobrado et al. [82] have recently reported that a burn unit contaminated with *A. baumannii *was successfully disinfected using an automated aerosolized hydrogen peroxide/silver cation dry-mist disinfection system.

**Iron chelation therapy**

Iron is an important cofactor in many processes occurring in bacterial cells; so, it is possible to view iron chelators and iron competitors as potential antibacterial agents. Chelation therapy is aimed at iron metabolism and achieving antibacterial activity by suppressing iron intake into cells. Pathogenic microorganisms have an effective mechanism for obtaining iron through using siderophores, low-molecular-weight compounds that bind iron [83]. The siderophore–iron complex binds to the corresponding receptors on the bacterial cell surface and is absorbed at places where iron is needed for intracellular metabolism. Most siderophores are high-affinity iron chelators whose affinity for Fe3+ is so high that they can use the host organism as a source of iron. Synthetic chelators have recently been developed to compete with the iron absorption systems of pathogenic microorganisms. The high efficiency of iron chelators (deferoxamine, deferiprone, Apo6619, and VK28) was evaluated against *A. baumannii *strains *in vitro *[84]. Synthetic iron chelators based on hydroxypyridinone ligands have been proposed as new bacteriostatic agents [83]. A number of new secondary/tertiary amine/amide chelators were obtained, and their antimicrobial properties were evaluated on the panel of microorganisms. Although it is an established fact that iron chelators can sequester iron and provide an alternative approach to treatment without the use of antibiotics, it is necessary to perform additional studies and characterize their *in vivo *effectiveness.

## CONCLUSIONS

Antibiotics can be viewed as chemical weapons in the interspecific struggle between microorganisms that has unfolded over millions of years of evolution. Moreover, each time a new antibiotic has been introduced into clinical practice, bacteria have developed an appropriate complex resistance strategy. As a result of this endless tug of war, pathogens armed with multiple resistance mechanisms have emerged, such as the *A. baumannii *considered in this review. The successful survival of *A. baumannii *as an in-hospital pathogen is facilitated by its high adaptability due to mutability and its ability to “switch” its genomic structure by horizontal transfer of resistance genes, as well as its innate ability to form biofilms.

The spread of multidrug-resistant strains necessitates the development of new approaches to the prevention and treatment of infections caused by *A. baumannii*, forcing us to search for alternative treatment methods that can be widely used in the future. The following methods have been proposed thus far: bacteriophage therapy, prophylactic vaccination, the use of antimicrobial peptides, photodynamic therapy, silver ion therapy, and chelate therapy. However, for each of these methods for preventing and treating the infections caused by MDR *A. baumannii*, there exist limitations that need to be addressed before these treatments can be applied in clinical practice.

In our review, we considered the existing research and prospects for expanding the means used to combat MDR *A. baumannii *strains.

## Abbreviations

WHO: World Health Organization
MDR: multidrug resistance
ESKAPE: Enterococcus faecium Staphylococcus aureus, Klebsiella pneumoniae, Acinetobacter baumannii, Pseudomonas aeruginosa, Enterobacter spp.
MIC: minimum inhibitory concentration
